# Who died as a result of the tsunami? – Risk factors of mortality among internally displaced persons in Sri Lanka: a retrospective cohort analysis

**DOI:** 10.1186/1471-2458-6-73

**Published:** 2006-03-20

**Authors:** Nobuyuki Nishikiori, Tomoko Abe, Dehiwala GM Costa, Samath D Dharmaratne, Osamu Kunii, Kazuhiko Moji

**Affiliations:** 1 Research Center for Tropical Infectious Diseases, Institute of Tropical Medicine, Nagasaki University, Nagasaki, Japan; 2 Department of Community Medicine, Faculty of Medicine, University of Peradeniya, Kandy, Sri Lanka

## Abstract

**Background:**

Describing adverse health effects and identifying vulnerable populations during and after a disaster are important aspects of any disaster relief operation. This study aimed to describe the mortality and related risk factors which affected the displaced population over a period of two and a half months after the 2004 Indian Ocean tsunami in an eastern coastal district of Sri Lanka.

**Methods:**

A cross-sectional household survey was conducted in 13 evacuation camps for internally displaced persons (IDP). Information on all pre-tsunami family members was collected from householders, and all deaths which occurred during the recall period (77 to 80 days starting from the day of the tsunami) were recorded. The distribution of mortality and associated risk factors were analysed. Logistic regression modelling using the generalized estimating equations method was applied in multivariate analysis.

**Results:**

Overall mortality rate out of 3,533 individuals from 859 households was 12.9% (446 deaths and 11 missing persons). The majority of the deaths occurred during and immediately after the disaster. A higher mortality was observed among females (17.5% vs. 8.2% for males, *p *< 0.001), children and the elderly (31.8%, 23.7% and 15.3% for children aged less than 5 years, children aged 5 to 9 years and adults over 50 years, respectively, compared with 7.4% for adults aged 20 to 29 years, *p *< 0.001). Other risk factors, such as being indoors at the time of the tsunami (13.8% vs. 5.9% outdoors, *p *< 0.001), the house destruction level (4.6%, 5.5% and 14.2% in increasing order of destruction, *p *< 0.001) and fishing as an occupation (15.4% vs. 11.2% for other occupations, *p *< 0.001) were also significantly associated with increased mortality. These correlations remained significant after adjusting for the confounding effects by multivariate analysis.

**Conclusion:**

A significantly high mortality was observed in women and children among the displaced population in the eastern coastal district of Sri Lanka who were examined by us. Reconstruction activities should take into consideration these changes in population structure.

## Background

On 26 December 2004, the largest earthquake of the past 40 years occurred off the west coast of northern Sumatra and generated the most deadly and devastating tsunami in recorded history [1,2]. Sri Lanka was the second most seriously affected country after Indonesia, with more than 31,000 deaths, 4,000 people missing, and half a million people displaced [3].

Understanding the impact of a disaster on human health is essential for effective disaster management. Epidemiological methods can be applied in disaster settings to describe the adverse health effects and to analyze the factors associated with them [4]. By identifying the vulnerable subgroups of a population, epidemiology can contribute to the effectiveness of interventions and to the appropriate allocation of resources.

Disaster epidemiology is a developing discipline, but reports have accumulated on various types of natural disasters such as earthquakes, floods, hurricanes, tornados and famines, as well as wars and other calamities of human origin. However, few epidemiological studies have been conducted on tsunamis, and their health consequences remain poorly documented.

We conducted a household survey to examine the mortality among internally displaced persons (IDP) during and after the Indian Ocean Tsunami in Sri Lanka.

While our previously published report described the temporal distribution of death, i.e. when people died [5], this study aimed at identifying the risk factors of the mortality. The study focused on the following question: "Who was most vulnerable during the tsunami disaster?"

## Methods

### Study design

The study was based on a cross-sectional household survey of the IDP conducted from 13 to 18 March 2005 in three administrative divisions of Ampara, an eastern coastal district of Sri Lanka. The mortality, the primary outcome measurement, was analysed as a retrospective cohort study.

The study protocol was reviewed and approved by the Ethical Review Committee, Faculty of Medicine, University of Peradeniya.

### Study population

The IDP were highly mobile, and some frequently relocated from one camp to another, especially when school buildings were vacated for the resumption of classes. In view of these difficulties in defining the sampling framework, we decided to include all camp residents in our study sample without any selection, i.e. all households in the 13 surveyed camps were invited to participate in the study.

### Survey

To define the household, we used a household registration card that was held by the householder. The local government issued the registration card for each affected family; it contains basic demographic information on all family members. The displaced people tended to keep the card securely because it was particularly important for receiving food rations and other aid supplies.

We invited all households in each camp to participate in the study and aimed at interviewing the householder as defined on the registration card. If the householder was not available for the interview, a proxy informant aged more than 15 years in the family (usually the householder's spouse) was invited. Either the householder or the proxy informant provided all information on the household and family members. There was no refusal among 859 invited households.

After giving written informed consent, the informants were interviewed by locally employed surveyors with a pre-piloted, structured questionnaire. Information on all family members as of 25 December 2004 (the day before the tsunami) was collected from the informant along with household information such as the area of residence, ethnicity, religion, occupation, household income and educational level. If any death occurred in the family between 26 December 2004 and the day of the survey, the informant was asked to recall the date and cause of the death. Other individual information included morbidity before the tsunami, age, sex, and the individual's location at the time of the tsunami.

To improve the accuracy of the demographic data, we verified the information on the family members by referring to the household registration card and the correction was made accordingly during the interview.

### Sample size

The sample size was calculated at 5% significant level and 90% power in order to detect the difference in mortality between 5% and 10% for males and females, based on the findings of our pilot study. Under the assumption of an intra-cluster (household) correlation of 0.5 and an average of four members per household, at least 777 households were needed for the analysis. Thus, we aimed at the collection of data on 800 households.

### Statistical analysis

The data were entered in a relational database developed on EpiData 3.1 (the EpiData Association, Denmark), in which a series of checking syntaxes was incorporated to detect data inconsistencies. The cleaned dataset was transferred to Stata 8.0 (Stata Corp., USA) for statistical analysis.

The sample was described initially by simple tabulations, followed by a crude analysis of the stratum-specific mortality and the odds ratio of death for each explanatory variable. The adjusted odds ratios were calculated by logistic regression modelling in order to control for the confounding effects between the explanatory variables. The variables were entered into the model one by one in order of strength of the effect on mortality. The model was evaluated at each addition of an explanatory variable by the likelihood ratio test against its preceding model. If the likelihood ratio test did not reveal any significant difference between the two models, the additional variable was removed from the model. Thus, the final model was established when the predictive capacity of the model would not improve further by entering any more variables.

Our individual observations were not likely to be independent because of the household-level sampling. In order to take into account the within-household correlation, the generalized estimating equations (GEE) method [6] was applied in the final logistic regression model.

## Results

### Sample characteristics

**Figure 1:**
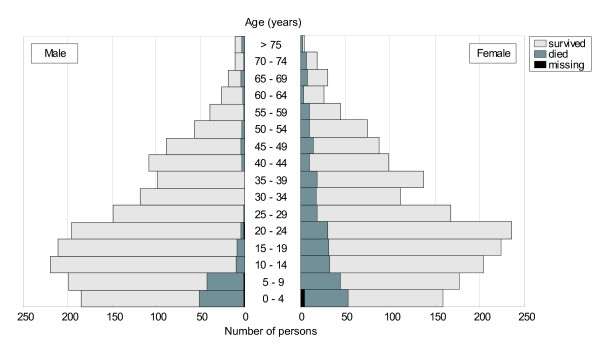
**Age/sex pyramid of 3,533 internally displaced persons due to the 2004 Indian Ocean tsunami in Ampara district, Sri Lanka**. The number of persons surviving, dead and missing by age group is shown for males and female separately.

The characteristics of the study sample are summarised in Table 1, with individual- and household-level variables treated separately. The sample consisted of 3,533 individuals, including 1,732 (49%) males, from 859 surveyed households. The detailed age distribution of the sample is presented as an age/sex pyramid in Figure 1. It shows a nearly symmetrical bell-shaped population structure and generally reflects the national population, which is characterised by a relatively large number of elderly people and fewer young children than might be expected for a South Asian country. The similarities between the population structure and the national statistics support the representative quality of our sample, at least demographically.

### Mortality

Overall, 446 deaths (12.6%) and 11 missing persons (0.3%) were reported. Despite the relatively long recall period (77 to 80 days), the mortality was concentrated within a few days after the disaster. Among 457 events of deaths or missing persons, 374 (82%) occurred on the day of the tsunami and the remaining 72 (18%) occurred in the following 7 days. No death was reported for more than two months thereafter [5]. Therefore, the following analysis for the risk factors should be interpreted as the risk factors for mortality during and immediately after the disaster. As missing persons were relatively few, we treated deaths and missing persons together in the subsequent statistical analysis.

**Figure 2:**
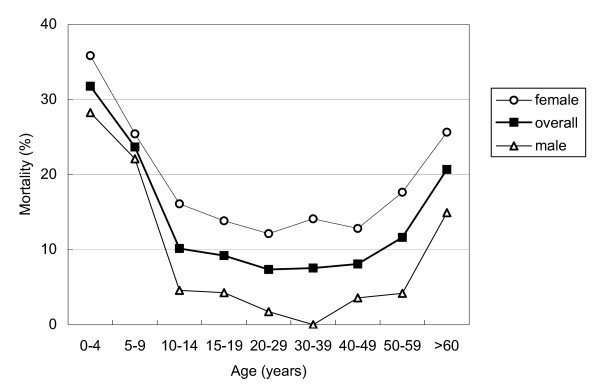
Age-band specific mortality among 3,533 internally displaced persons due to the 2004 Indian Ocean tsunami in Ampara district, Sri Lanka.

The age-specific mortality is presented for males and females separately in Figure 2. A strikingly high mortality was observed among children and elderly people. Also, the female population showed a consistently higher mortality than the male population across all age groups.

### Risk factors

In order to identify the risk factors of mortality, the stratum-specific mortality and the crude odds ratio for each explanatory variable were calculated, as shown in Table 2. Again, young children and the oldest age group faced significantly higher odds of death compared to people in their twenties (OR (odds ratio) (95% CI (confidence interval)) = 5.87 (4.03–8.55) for children aged less than 5 years, 3.91 (2.69–5.68) for children aged 5 to 9 years and 2.27 (1.52–3.39) for adults over 50 years old). The female population showed a significantly higher mortality than the male, with an OR (CI) of 2.37 (1.92–2.94).

The location of individuals at the time of the tsunami was significantly associated with mortality. In general, individuals inside buildings had a significantly higher mortality than those outdoors at the time. Even compared with those who were on the beach or in the sea, people who were at home were more likely to die. Pre-existing health problems, the area of residence, the living period and the ethnic background were not associated with mortality.

The mortality tended to decrease the higher the educational level (OR = 1.00, 0.84 and 0.41 for primary, secondary and higher education, respectively), but this result did not apply to households with no school education, which showed low mortality. Among the various categories of occupation, only fishing showed a significantly higher mortality than the other occupations (OR = 1.44 (1.18–1.75) compare to all other occupations). Although the household income varied from 0 to more than 15,000 rupees (approximately 150 USD) per month, there was no evidence of an association between income and mortality. As expected, the level of house destruction correlated linearly with mortality; a significantly high mortality was observed among people whose houses were totally destroyed as compared to the minimum destruction level (OR (CI) = 3.43 (1.74–6.75)).

It is likely that some of the identified risk factors were interrelated. For example, the increased risk associated with being indoors may have been confounded by age and sex because women and children were more likely to be in their houses on the morning of the tsunami. In order to control for the confounding effects between the explanatory variables, the adjusted odds ratios were obtained by a logistic regression model according to the procedure described in the previous section. For two variables, the location at the time of the tsunami and the occupation of the householder, the categories were aggregated into dichotomous values for the purpose of modelling. The final model included age, sex, location at the time of the tsunami, educational level, occupation, and house destruction level. The GEE method was applied to the final model to account for within-household correlation. As shown in Table 2, the adjusted odds ratios were broadly similar to the corresponding crude odds ratios, and all variables entered in the model continued to be risk factors of mortality without a substantial reduction in the odds ratios.

In conclusion, women, children and the elderly were found to comprise the most vulnerable population. The location indoors and the level of house destruction were also significantly associated with mortality. Among the socio-economic measurements, fishing as the household occupation and the educational level of householders showed some evidence of association while, notably, household income was not associated with mortality.

## Discussion

The limitations of our study include potential selection bias and the lack of a population-based estimate due to the sampling from IDP camps. Although we identified all pre-tsunami family members by interviewing the householders, we examined only the households that had been accommodated in the IDP camps. Thus, the mortality presented in this study is probably higher than the population-based statistics that include people who did not take refuge in the IDP camps but stayed in their own houses after the tsunami, which obviously would have suffered less destruction.

It is also possible that our sample population is an even more vulnerable group among all of the IDP affected by the tsunami. This is because by the time the survey was conducted, relatively affluent families might have moved out from IDP camps and resettled in other locations such as a relative's house. If it is the case, the variation of the exposure measurements were likely to be reduced in our study sample, potentially leading to the decreased effects of the risk factors on mortality. Nevertheless, considering the fact that our sample preserved its population structure representing the national statistics, we believe that our study population is still useful for identifying the risk factors of mortality especially for demographic factors.

We observed unexpected findings for some socio-economic variables. Decreased mortality was observed among families with the lowest educational level and apparently there was no association between household income levels and mortality. It has been generally recognized that various socio-economical indicators are the important determinants in predicting who are the most vulnerable during disasters. A question may be raised as to whether we can conclude that our results are inconsistent with this general perception; possibly reflecting the non-discriminative nature of the tsunami disaster. However, we cannot draw a conclusive statement from our study because of the limitations set-out below.

There might have been some residual confounding effects behind some of the associations which were not totally controlled. For example, the families in the lowest educational category tended to have a higher age distribution of the family members, which probably reflects the long-term improvement of school enrolment rate over the past decades in Sri Lanka. Because high mortality was observed among young children aged less than 10 years, families whose members were older in age and often without small children, might have experienced decreased mortality.

Although we have conducted a multivariate analysis, it is possible that the confounding effect by age was not totally controlled because of the difference in the measurement level (educational level was measured at household level while age at individual level).

Another important limitation could be the lack of enough variation in exposures due to the sampling from possibly the most vulnerable IDP as noted above. Although the income level ranged from zero to more than 15,000, the majority falls into the categories of 1–2999 and 3000–5999 Sri Lankan Rupees and few households were in the highest category. This distribution of income level seems low comparing previous available data. According to the Household Income and Expenditure Survey 2002/3 by Department of Census and Statistics, the median and mean household income for the Eastern Province (where our study area is located) were 5500 and 7640 respectively and around 20% of the sample households reported more than 10,000 Sri Lankan Rupees of income.

Therefore, it is possible that our sample population was a somewhat homogeneous population from relatively low socio-economic groups. This meant that the effect of socio-economic indicators on mortality did not appear to be substantial, i.e. there were reduced effects due to the lack of variation in exposures. Considering these points, our study could not provide a decisive conclusion on the correlation between some socio-economic indicators and mortality.

This study revealed a significantly high mortality rate in women, children and the elderly. A similar gender-age mortality pattern has been reported in other disaster settings. In both the 1999 Taiwan earthquake [7,8] and the 1988 Armenian earthquake [9], the elder population was particularly at risk of death [10], and there was a high mortality rate among women and young children. In the 1995 Great Hanshin-Awaji Earthquake in Japan, a significantly high mortality rate was reported among the elderly, especially those with physical disabilities [11].

By contrast, previous studies on floods have revealed clearly different mortality patterns. In an extensive study on flood-related deaths in Europe and the United States, middle-aged men were found to be the most vulnerable population [12]. A study in Australia also reported that 80% of flood deaths occurred among the male population [13]. Both studies suggested that risky behaviour, such as trying to swim across rivers or using motor vehicles to flee, resulted in the increased mortality of this population.

The findings of our study indicate that the age-gender mortality pattern of the tsunami victims is similar to that of earthquakes but very different to that of floods. This clear difference in the high-risk groups observed in different types of disasters highlights the need for epidemiological studies to precisely identify the vulnerable groups unique to each disaster setting, and to provide information for subsequent disaster management and planning. We believe that our study contributes to the epidemiological evidence on tsunami-related mortality, which has not been well described to date.

In planning reconstruction aid activities, the impact of the change in population structure on the affected community should be thoroughly considered. The loss of the middle-aged female population may have a particularly significant impact, because these individuals are the primary caretakers of their children and families, as highlighted by the officials from WHO and UNICEF [14]. The nutritional status and morbidity among the surviving children, including psychological illness, should be carefully monitored. The incidence of certain conditions among the surviving adult population, especially cardiovascular disease, as already noted in our study population [5], or mental disorders including suicidal tendencies, demands further attention because the long-term risks of these conditions have been pointed out in other disaster settings [15-17]. Although not imminent, social instability and the danger of an increase in sexually transmitted diseases, including HIV/AIDS, also deserve attention because the male-female ratio increased from 97.8:100 to 108.3:100 (before and after the tsunami) among people under 50 years of age in our study sample.

The consequences of disasters are very complex. Disaster epidemiology can play an important role in describing the adverse health effects and identifying the vulnerable groups during and after disasters. It enables relief and reconstruction activities to be more focused, relevant and efficient. Continuing research is needed to monitor and evaluate the long-term impact of the tsunami disaster on human health.

## Conclusion

A significantly high mortality was observed in women and children among the displaced population of the eastern coastal district of Sri Lanka who were examined by us. The loss of the middle-aged female population may pose further risks of morbidity, including malnutrition, in the surviving children. Aid agencies and the government may need to take the change in population structure into account in order to deliver reconstruction aid which is relevant to the local social situation and context.

## Competing interests

The author(s) declare that they have no competing interests.

## Authors' contributions

TA, NN and OK designed and coordinated the study and NN is the guarantor. TA, DGMC and SDD conducted and supervised the filed study. TA, NN and KM analysed and interpreted the data. NN developed the draft and the revisions were made by all authors. All authors approved the final version.

## Pre-publication history

The pre-publication history for this paper can be accessed here:



## Figures and Tables

**Table 1:** Characteristics of study sample of 3,533 individuals from 859 displaced households in Ampara, Sri Lanka due to the 2004 Indian Ocean tsunami.

		**N**	**(%)**
**Individual level**	**Total**	**3,533**	

Sex	male	1732	49.0%
Location when tsunami hit	indoors	3,128	88.5%
	outdoors	405	11.5%
Pre-existing health problem	yes	339	9.6%
Status	survived	3,076	87.1%
	died	446	12.6%
	missing	11	0.3%

**Household level**	**Total**	**859**	

Living area	Karaitivu	475	55.3%
	Sainthamarthu	217	25.3%
	Kalmunai	167	19.4%
Ethnicity	Shinhalese	2	0.2%
	Tamil	474	55.2%
	Moor	383	44.6%
Educational level (highest level among householder and spouse)	no school	83	9.7%
	primary	198	23.1%
	secondary	513	59.7%
	higher	65	7.6%
Occupation of householder	farming	30	3.5%
	fishing	318	37.0%
	laborer	274	31.9%
	trader	35	4.1%
	professional	29	3.4%
	others	168	19.6%
	none	5	0.6%
Household income (Rupee)	nothing	49	5.7%
	-2999	308	35.9%
	3000-	309	36.0%
	6000-	127	14.8%
	10000-	52	6.1%
	15000-	14	1.6%
House destruction	less than half	48	5.6%
	more than half	73	8.5%
	totally	738	85.9%

**Table 2:** Mortality, odds ratios (OR) and adjusted odds ratios (AOR) among internally displaced persons in Ampara, Sri Lanka due to the 2004 Indian Ocean tsunami.

		**N**	**mortality**	**OR (95% CI)**	**AOR (95% CI)**
**Total**		3,533	12.9%		

**Individual level**					
Age (years)	<5	343	31.8%	5.87 (4.03–8.55)***	5.90 (4.21–8.26)***
	5–9	376	23.7%	3.91 (2.69–5.68)***	3.94 (2.74–5.66)***
	10–14	424	10.1%	1.42 (0.94–2.16)	1.41 (0.92–2.16)
	15–19	435	9.2%	1.28 (0.83–1.95)	1.14 (0.75–1.74)
	20–29	748	7.4%	1.00	1.00
	30–39	464	7.5%	1.03 (0.66–1.60)	0.97 (0.63–1.49)
	40–49	383	8.1%	1.11 (0.70–1.76)	1.18 (0.75–1.85)
	>50	360	15.3%	2.27 (1.52–3.39)***	2.53 (1.62–3.96)***
Sex	Male	1,732	8.2%	1.00	1.00
	Female	1,801	17.5%	2.37 (1.92–2.94)***	2.79 (2.29–3.39)***
Location when tsunami hit	own house	3,042	13.8%	1.00	
	someone's house	31	22.6%	1.82 (0.78–4.25)	
	mosque/temple	3	0.0%	0.00	
	other inside	52	11.5%	0.81 (0.35–1.92)	
	around own house	204	7.4%	0.50 (0.29–0.85)**	
	Beach	32	9.4%	0.65 (0.20–2.13)	
	Sea	36	8.3%	0.57 (0.17–1.86)	
	other outside	133	2.3%	0.14 (0.05–0.46)***	
	inside total	3,128	13.8%	2.55 (1.67–3.91)***	2.87 (1.44–5.71)**
	Outside total	405	5.9%	1.00	1.00
Pre-existing health problem	No	3,194	12.8%	1.00	
	Yes	339	14.2%	1.12 (0.81–1.55)	

**Household level**					
Living area	Karaitivu	1,847	12.1%	1.00	
	Sainthamarthu	986	14.0%	1.19 (0.94–1.49)	
	Kalmunai	801	13.5%	1.13 (0.89–1.45)	
Living periods (years)	0–9	661	13.6%	1.00	
	10–19	775	15.2%	1.14 (0.85–1.53)	
	20–29	1,095	12.0%	0.86 (0.65–1.15)	
	>30	994	11.9%	0.85 (0.64–1.15)	
Ethnicity	Shinhalese	10	10.0%	0.80 (0.10–6.36)	
	Tamil	1,806	12.2%	1.00	
	Muslim	1,717	13.7%	1.15 (0.94–1.40)	
Educational level	no school	260	7.7%	0.46 (0.28–0.75)**	0.41 (0.20–0.88)*
	Primary	887	15.3%	1.00	1.00
	secondary	2,141	13.3%	0.84 (0.68–1.05)	0.90 (0.65–1.25)
	Higher	245	6.9%	0.41 (0.24–0.70)***	0.48 (0.24–0.95)*
Occupation	Farming	127	9.5%	1.00	
	Fishing	1,433	15.4%	1.75 (0.95–3.22)	
	Laborer	1,122	10.3%	1.11 (0.59–2.06)	
	Trader	155	10.3%	1.10 (0.50–2.43)	
	professional	122	6.6%	0.67 (0.26–1.71)	
	Others	574	14.6%	1.64 (0.87–3.11)	
	Fishing	1,433	15.4%	1.44 (1.18–1.75)***	1.38 (1.04–1.85)*
	Others	2,100	11.2%	1.00	1.00
Household income (Rupee)	Nothing	132	12.9%	1.00	
	1–2999	1,221	12.0%	0.93 (0.54–1.59)	
	3000-	1,348	13.4%	1.05 (0.62–1.79)	
	6000-	567	12.5%	0.97 (0.55–1.71)	
	10000-	210	16.2%	1.31 (0.70–2.45)	
	15000-	55	12.7%	0.99 (0.38–2.54)	
Level of house destruction	less than half	195	4.6%	1.00	1.00
	more than half	308	5.5%	1.21 (0.53–2.77)	1.52 (0.51–4.55)
	Totally	3,030	14.2%	3.43 (1.74–6.75)***	3.79 (1.49–9.66)**

* p < 0.05, ** p < 0.01, *** p < 0.001

CI; confidence interval

† Adjusted odds ratios were obtained by a logistic regression model, containing variables for age band, sex, location when tsunami hit, educational level, fishing as an occupation and the house destruction level.
